# Supplementary material for: Hummingbird plumage color diversity exceeds the known gamut of all other birds
Source: Commun Biol. 2022 Jun 23;5:576. doi: 10.1038/s42003-022-03518-2 (PMC9226176; doi:10.1038/s42003-022-03518-2)
Supplement: Supplementary file 2 — Supplementary Information [file 42003_2022_3518_MOESM2_ESM.pdf]

Hummingbird plumage color diversity exceeds the known gamut for all other birds

SUPPLEMENTARY TABLES AND FIGURES

Supplementary Tables

Supplementary Table 1: Means, standard deviations (St.dev), and coefficients of variation (CV) in hummingbird plumage color gamut size for the resampled datasets (n = 10 datasets) according to number of species (sps) resampled.

	60 sps	70 sps	80 sps	90 sps	100 sps	110 sps
Mean	0.0627	0.0640	0.0658	0.0711	0.0718	0.0723
St.dev	0.0057	0.0067	0.0061	0.0034	0.0032	0.0033
CV	0.0908	0.1052	0.0934	0.0477	0.0450	0.0455

Supplementary Table 2: Summary statistics describing both the total UVS avian plumage gamut and the total UVS hummingbird gamut.

	# of measurements	Volume	% of Color Space	% of Avian Gamut	Average Color span	Max span	Average Hue disp	Max hue disp	Average chroma
**Hummingbird**									
All	5000	6.42E-02	29.6	62.6	2.33E-01	8.58E-01	1.04E+00	3.14E+00	2.11E-01
Struc. Barb.	3376	6.17E-02	28.5	60.2	2.67E-01	8.58E-01	1.11E+00	3.14E+00	2.51E-01
Melanins	1189	3.20E-03	1.48	3.12	1.24E-01	5.00E-01	6.42E-01	3.11E+00	1.34E-01
Eumelanins	824	1.71E-03	0.790	1.67	8.46E-02	3.64E-01	7.70E-01	3.11E+00	8.81E-02
Phaomelanins	365	1.73E-03	0.801	1.69	9.97E-02	4.41E-01	2.69E-01	1.39E+00	2.38E-01
Whites	435	8.37E-05	0.0387	0.0817	4.54E-02	2.09E-01	1.56E-01	9.34E-01	1.10E-01
**Total Aves**									
S&P	966	6.50E-02	30.0	63.4	2.13E-01	9.50E-01	1.29E+00	3.14E+00	1.48E-01
S&P & Humm.	5966	1.02E-01	47.3	100	2.35E-01	9.53E-01	1.10E+00	3.14E+00	2.01E-01
S&P Struc. Barb.	98	2.27E-02	10.5	22.1	1.87E-01	5.52E-01	1.26E+00	3.09E+00	1.42E-01
S&P & Humm. Struc. Barb.	3474	6.69E-02	30.9	65.3	2.66E-01	8.58E-01	1.11E+00	3.14E+00	2.48E-01

S&P" refers to Stoddard and Prum[2] 's results while" S&P & Humm." refers to the combination of our hummingbird data and their data. "Struc. Barb." refers to structural barbules

Supplementary Figures

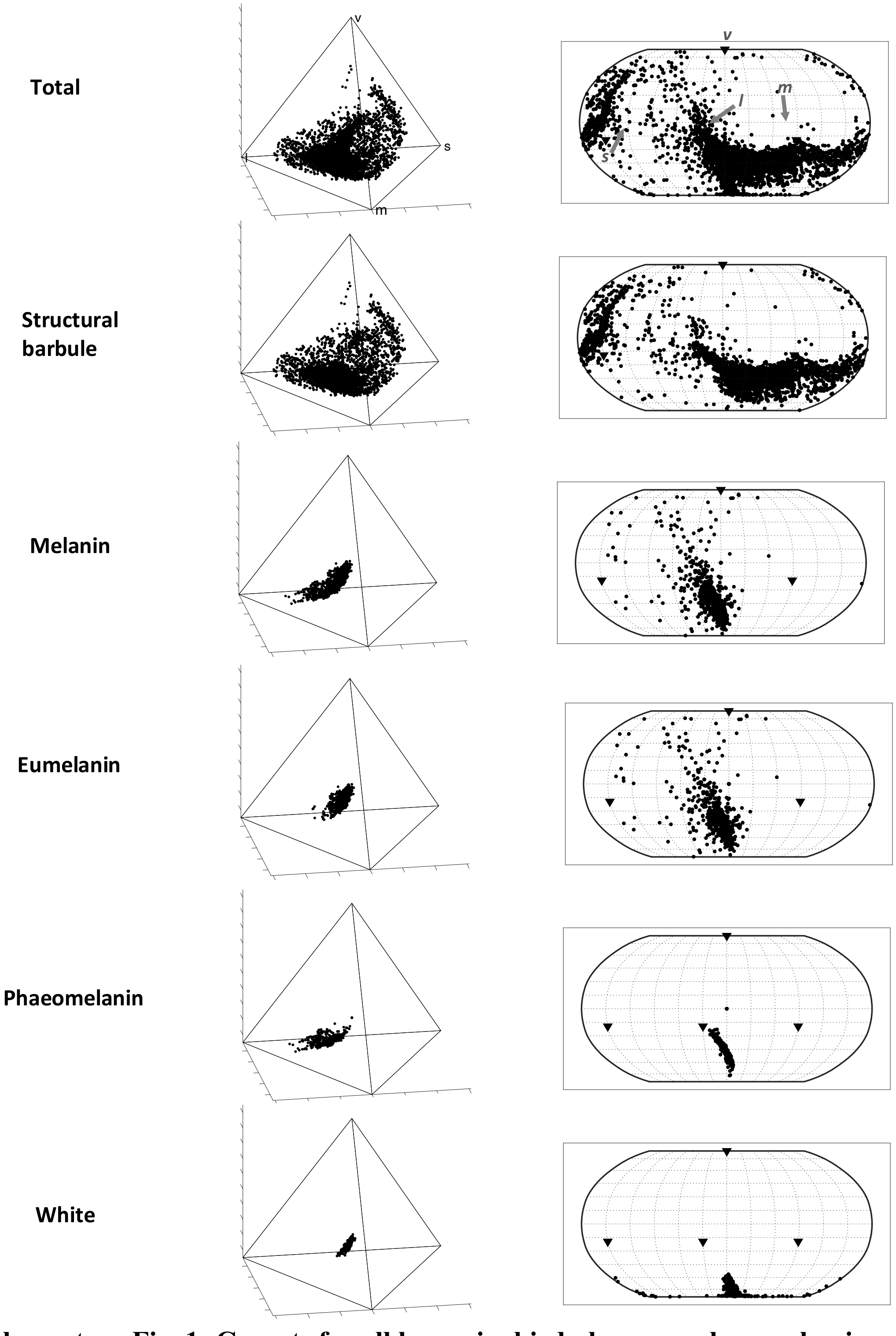

Supplementary Fig. 1: Gamuts for all hummingbird plumage color mechanisms shown in an avian VS colorspace. Tetrahedron plots (left) and corresponding Robinson Projections (right).

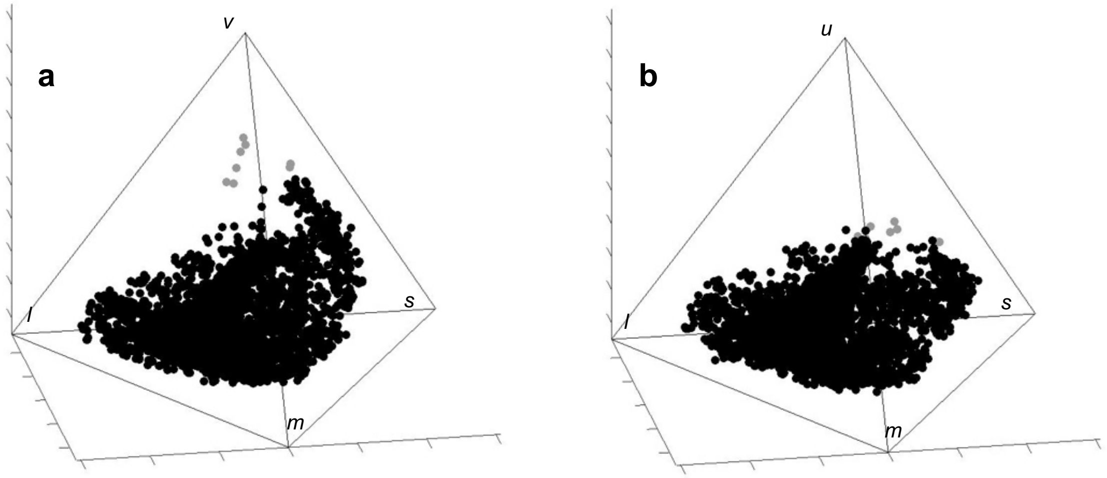

Supplementary Fig. 2: Comparison of hummingbird plumage gamut as seen by the VS-type and the UVS-type visual systems. Grey spectra (gorgets of *Boissonneaua jardini* and *Heliangelus viola*) map closer to the *v* vertex in **a** the VS-type visual system than to the *uv* vertex in **b** the UVS-type visual system.

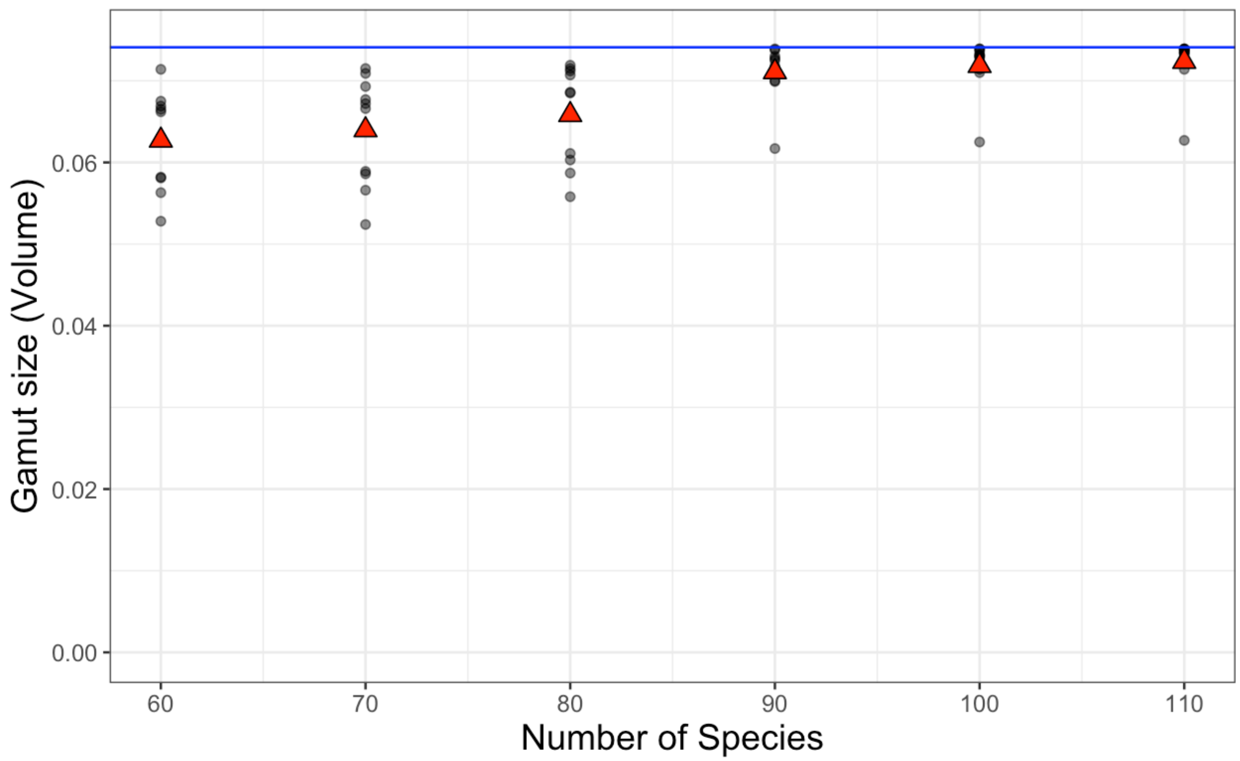

Supplementary Fig. 3: The relationship between the number of species included in a dataset and hummingbird plumage color gamut size. The red triangles are the means of the gamut size of the ten resampled datasets corresponding to that number of species, which are the black points. The blue line shows the volume we calculated with our total hummingbird data. All gamut volumes below the mean for a given number of species were in samples that excluded *Boissonneaua jardini.* Values for the means, standard deviations, and coefficients of variation are located in Supplementary Table 1.

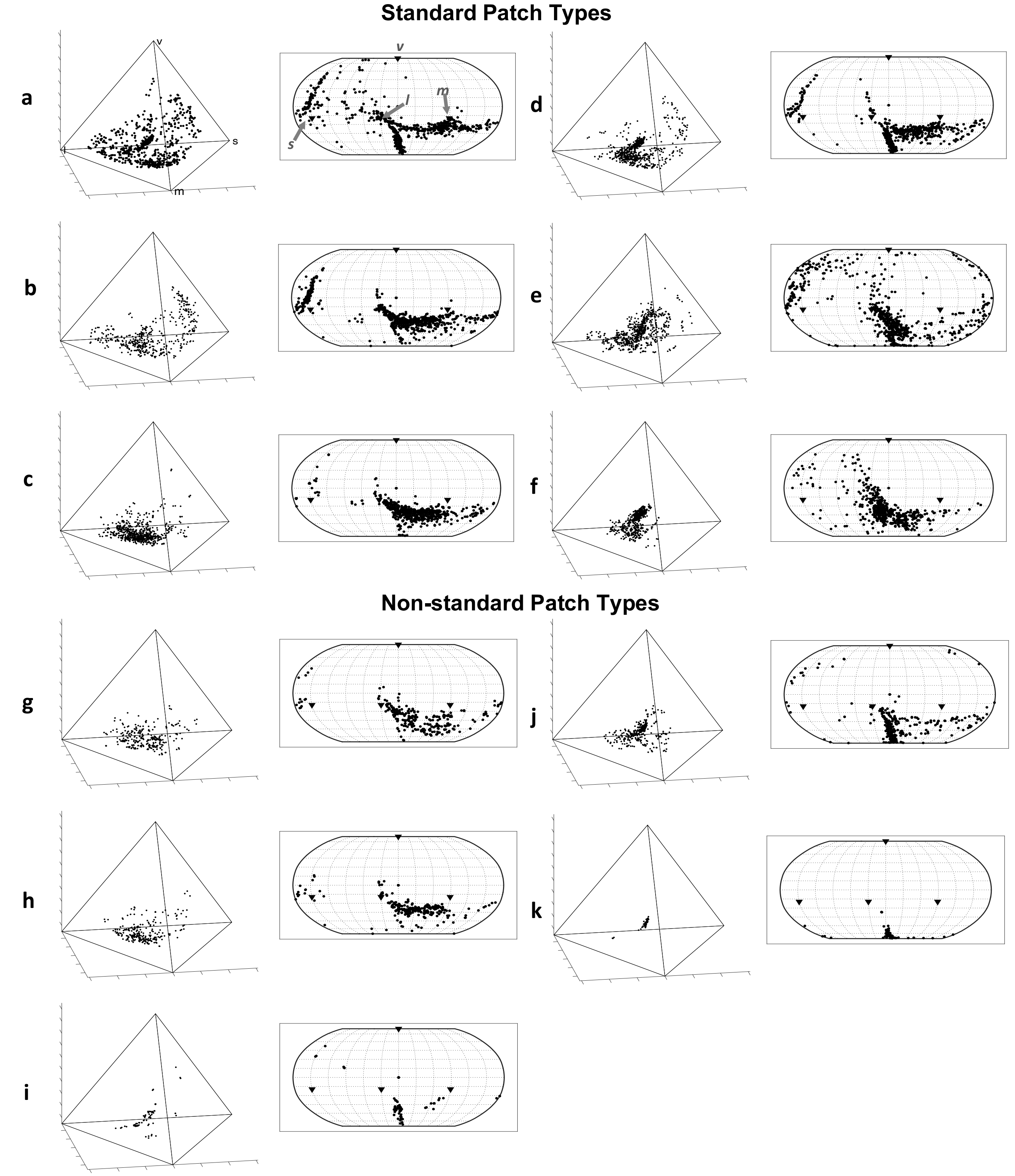

Supplementary Fig. 4: Gamuts for all hummingbird body patch types shown in an avian VS colorspace. Tetrahedron plots (left) and corresponding Robinson Projections (right). **a-f** Standard patch types included **a** throat, **b** crown, **c** back, **d** belly, **e** tail, and **f** wing. **g-i** Nonstandard patches included **g** rump, **h** nape, **i** cheek, **j** undertail coverts, and **k** legs.

Supplementary Figure 5 available at: doi:10.5061/dryad.1c59zw3xn

Supplementary Fig. 5: Gamuts for all examined hummingbird species shown in an avian VS color space. Spectra mapped in a tetrahedron color space from a standard view and a top down view as well as in a Robinson projection. Figure available at: doi:10.5061/dryad.1c59zw3xn.
