# Supplementary material for: Hummingbird plumage color diversity exceeds the known gamut of all other birds
Source: Commun Biol. 2022 Jun 23;5:576. doi: 10.1038/s42003-022-03518-2 (PMC9226176; doi:10.1038/s42003-022-03518-2)
Supplement: Supplementary file 3 — Description of Additional Supplementary Files [file 42003_2022_3518_MOESM3_ESM.pdf]

Description of Additional Supplementary Files

File name: Supplementary Data 1

Description: Species summary statistics describing the gamut size and the distribution of plumage colors. Species are arranged in phylogenetic order according largely to McGuire et al.31. An asterisk is placed next to the species names of all species for which we missed at least one patch measurement. Color span describes the Euclidean distances between the species‟ color points, hue disparity describes the magnitude of the angles between color vectors, and chroma (i.e. saturation) is the distance of a color point from the achromatic origin.
