# Supplementary material for: Hummingbird plumage color diversity exceeds the known gamut of all other birds
Source: Commun Biol. 2022 Jun 23;5:576. doi: 10.1038/s42003-022-03518-2 (PMC9226176; doi:10.1038/s42003-022-03518-2)
Supplement: Supplementary file 5 — Reporting Summary [file 42003_2022_3518_MOESM5_ESM.pdf]

nature research

Corresponding author(s): Gabriela X. Venable
Richard O. Prum

Last updated by author(s): Mar 28, 2022

Reporting Summary

Nature Research wishes to improve the reproducibility of the work that we publish. This form provides structure for consistency and transparency in reporting. For further information on Nature Research policies, see our Editorial Policies and the Editorial Policy Checklist.

Statistics

For all statistical analyses, confirm that the following items are present in the figure legend, table legend, main text, or Methods section.

n/a	Confirmed	
☐	☒	The exact sample size (*n*) for each experimental group/condition, given as a discrete number and unit of measurement
☐	☒	A statement on whether measurements were taken from distinct samples or whether the same sample was measured repeatedly
☐	☒	The statistical test(s) used AND whether they are one- or two-sided<br>*Only common tests should be described solely by name; describe more complex techniques in the Methods section.*
☒	☐	A description of all covariates tested
☐	☒	A description of any assumptions or corrections, such as tests of normality and adjustment for multiple comparisons
☐	☒	A full description of the statistical parameters including central tendency (e.g. means) or other basic estimates (e.g. regression coefficient) AND variation (e.g. standard deviation) or associated estimates of uncertainty (e.g. confidence intervals)
☒	☐	For null hypothesis testing, the test statistic (e.g. F, t, r) with confidence intervals, effect sizes, degrees of freedom and P value noted<br>*Give P values as exact values whenever suitable.*
☒	☐	For Bayesian analysis, information on the choice of priors and Markov chain Monte Carlo settings
☒	☐	For hierarchical and complex designs, identification of the appropriate level for tests and full reporting of outcomes
☒	☐	Estimates of effect sizes (e.g. Cohen's *d*, Pearson's *r*), indicating how they were calculated

Our web collection on statistics for biologists contains articles on many of the points above.

Software and code

Policy information about availability of computer code

Data collection	We used an R-script to compile all of our data from separate reflectance spectrum TXT files into CSV files with all the data collected for each species. This R-script is available at doi:10.5061/dryad.1c59zw3xn.
Data analysis	TetraColorSpace software available online at https://www.marycstoddard.com/software

For manuscripts utilizing custom algorithms or software that are central to the research but not yet described in published literature, software must be made available to editors and reviewers. We strongly encourage code deposition in a community repository (e.g. GitHub). See the Nature Research guidelines for submitting code & software for further information.

Data

Policy information about availability of data

All manuscripts must include a data availability statement. This statement should provide the following information, where applicable:

- Accession codes, unique identifiers, or web links for publicly available datasets
- A list of figures that have associated raw data
- A description of any restrictions on data availability

The data depicted by the figures and tables are available on Dryad (doi:10.5061/dryad.1c59zw3xn). Individual spectrum TXT files for each measurement are also available from the corresponding author on reasonable request. Skin specimens were from the Yale Peabody Museum and the American Museum of Natural History (Catalogue numbers located in Supplementary Data 1). The data from Stoddard and Prum 20112 are available at doi:10.1093 /beheco/arr088).

Field-specific reporting

Please select the one below that is the best fit for your research. If you are not sure, read the appropriate sections before making your selection.

☐ Life sciences ☐ Behavioural & social sciences ☒ Ecological, evolutionary & environmental sciences

For a reference copy of the document with all sections, see nature.com/documents/nr-reporting-summary-flat.pdf

Ecological, evolutionary & environmental sciences study design

All studies must disclose on these points even when the disclosure is negative.

Study description	We examined the diversity of plumage color of the hummingbird family (114 species and 5,000 spectra) and quantified it using a avian vision tetrahedron color space model. A common set of body patches was measured for each species and additional patches were measured if they were distinct to the human eye and large enough to measure reliably. Spectra were mapped as color points and the diversity of a taxon was defined as the minimum convex multi-dimensional volume that covers those color points. This volume is a unitless measure, so no units were given. This volume was then compared to that previously known for all birds.
Research sample	Spectra were collected from study skin specimens from the Yale Peabody Museum (YPM) and the American Museum of Natural History (AMNH). This sample reflected about 33% of all extant hummingbird species. To maximize the estimated hummingbird plumage color gamut, species were selected for unique colors or diverse plumages. To represent the diversity of the family, some species were included from each major clade regardless of coloration. Mostly male specimens were used as they are usually the most colorful sex. The data for the previously known total avian color diversity came from a 2011 paper.
Sampling strategy	Following the methods of a previous paper, reflectance spectra were measured from six standardized patches from all specimens: crown, back, tail, wing, belly, and throat. Additional patches were measured if they were distinct to the human eye and large enough to measure reliably. Each plumage patch was measured from a different position three times. Multiple measurements were not averaged to prevent flattening of highly saturated peaks with slightly different hues. If a patch showed a gradient in color, measurements were taken at the ends and center of the gradient. The sample size reflects the total number of colors distinguishable by the eye for each species.
Data collection	Reflectance spectra were measured by the first author. They used a S2000 Ocean Optics spectrometer and a bifurcated fiber with an Ocean Optics DH-2000-BAL deuterium–halogen light source (Ocean Optics, Dunedin, FL) in a dark room with an integration time of 100 ms. We did not use a metal block to hold the reflectance probe because it can be difficult to accurately measure plumage reflectance peaks from small iridescent patches at a normal angle of incidence to the plumage surface. Rather, we used a Keysight 3D Probe Positioner to hold the optical fiber stable at the appropriate angle of incidence to maximize peak reflectance and saturation while maintaining peak reflectance below 100%. This method allowed us to preserve some within patch color variation while also increasing repeatability of spectra from iridescent structural colored patches, because it allowed us to find the appropriate angle that produced the most saturated spectra: The most saturated spectra exhibit normal incidence to the laminar nanostructures in the barbules, which may not be normal to the plane of the plumage surface.
Timing and spatial scale	These data were collected from June 2019 to April 2020. No specific frequency /periodocity was used because this data is not time sensitive.
Data exclusions	Data from presumed structural black plumage were were excluded from out analyses because their extremely low reflectance (< 5% reflectance at all distances) caused irregular mapping in some of our analyses
Reproducibility	We used a Keysight 3D Probe Positioner to hold the optical fiber stable at the appropriate angle of incidence to maximize peak reflectance and saturation while maintaining peak reflectance below 100%. This method allowed us to preserve some within patch color variation while also increasing repeatability of spectra from iridescent structural colored patches, because it allowed us to find the appropriate angle that produced the most saturated spectra: The most saturated spectra exhibit normal incidence to the laminar nanostructures in the barbules, which may not be normal to the plane of the plumage surface.
Randomization	Spectra were labeled with presumed coloration mechanisms. Color production mechanisms were inferred based on previous literature, visual appearance, and the shape of the reflectance spectra. Any color that showed barbule structural color as well as melanin was categorized as a barbule structural color.
Blinding	Blinding was not appropriate for this study because we were specifically looking to document hummingbird color diversity, which involves the selection and measurement of species that have unique plumage colors.

Did the study involve field work? ☐ Yes ☒ No

Reporting for specific materials, systems and methods

We require information from authors about some types of materials, experimental systems and methods used in many studies. Here, indicate whether each material, system or method listed is relevant to your study. If you are not sure if a list item applies to your research, read the appropriate section before selecting a response.

Materials & experimental systems

n/a	Involved in the study	
☒	☐	Antibodies
☒	☐	Eukaryotic cell lines
☒	☐	Palaeontology and archaeology
☐	☒	Animals and other organisms
☒	☐	Human research participants
☒	☐	Clinical data
☒	☐	Dual use research of concern

Methods

n/a	Involved in the study	
☒	☐	ChIP-seq
☒	☐	Flow cytometry
☒	☐	MRI-based neuroimaging

Animals and other organisms

Policy information about studies involving animals; ARRIVE guidelines recommended for reporting animal research

Laboratory animals	NA
Wild animals	NA
Field-collected samples	This study involved museum study specimens.
Ethics oversight	No ethical approval was necessary because we were using study skin specimens.

Note that full information on the approval of the study protocol must also be provided in the manuscript.
